# Knowledge and periconceptional use of folic acid for the prevention of neural tube defects in ethnic communities in the United Kingdom: Systematic review and meta-analysis

**DOI:** 10.1002/bdra.23154

**Published:** 2013-07-19

**Authors:** Jordana N Peake, Andrew J Copp, Jill Shawe

**Affiliations:** 1 Institute for Women’s Health, University College LondonUnited Kingdom; 2 Institute of Child Health, University College LondonUnited Kingdom

**Keywords:** neural tube defects, ethnicity, folic acid, prevention, preconception, periconception

## Abstract

**BACKGROUND:** It is widely accepted that periconceptional supplementation with folic acid can prevent a significant proportion of neural tube defects (NTDs). The present study evaluated how folic acid knowledge and periconceptional use for NTD prevention varies by ethnicity in the United Kingdom (U.K.).

**METHODS:** A literature search was conducted to identify studies that included assessment of folic acid knowledge or use in U.K. women of different ethnicities. Only research and referenced sources published after 1991, the year of the landmark Medical Research Council’s Vitamin Study, were included. A meta-analysis was performed of studies that assessed preconceptional folic acid use in Caucasians and non-Caucasians.

**RESULTS:** Five studies met the inclusion criteria for assessment of knowledge and/or use of folic acid supplements in U.K. women including non-Caucasians. The available evidence indicates that South Asians specifically have less knowledge and lower periconceptional use of folic acid than Caucasians; one study found that West Indian and African women also had lower folic acid uptake. A synthesis of results from three of the studies, in a meta-analysis, shows that Caucasians are almost three times more likely to take folic acid before conception than non-Caucasians.

**CONCLUSION:** From the limited evidence available, U.K. women of non-Caucasian ethnicity appear to have less knowledge and a lower uptake of folic acid supplementation than Caucasians during the periconceptional period. Implementing targeted, innovative education campaigns together with a mandatory fortification policy, including the fortification of ethnic minority foods, will be required for maximum prevention of folic acid–preventable NTDs across different ethnic groups. *Birth Defects Research (Part A) 97:444–451, 2013*. © 2013 Wiley Periodicals, Inc.

## INTRODUCTION

Neural tube defects (NTDs) are congenital abnormalities caused by failed closure of the embryonic neural tube. Closure is normally complete by day 28 of pregnancy, before many women know they are pregnant. NTDs affect 0.5–2 in every 1000 pregnancies worldwide (Greene at al., 2009), with a prevalence in the United Kingdom (U.K.) estimated to be 1 per 1000 births (EUROCAT, 2009).

The 1991 international Medical Research Council double blind randomized controlled trial was the first to unambiguously show a link between folic acid supplementation and NTD reduction. There was a 72% reduction in NTD recurrence in women taking folic acid supplements (MRC Vitamin Study Research Group, 1991). It was argued by the authors that although the trial assessed NTD reduction in those with a previous NTD-affected pregnancy, there was no reason to expect that folic acid would not have the same effect on first occurrence NTDs (MRC Vitamin Study Research Group, 1991). Indeed, a subsequent Hungarian randomized clinical trial confirmed that folic acid also has a protective effect for first occurrence NTDs (Czeizel and Dudas, 1992). It has been estimated that the percentage of NTDs that are folate sensitive globally is 75% (Bell and Oakley, 2009) and, for the United States, 50% (CDC, 1992).

The 1992 Centers for Disease Control and Prevention recommendation of 400 μg daily folic acid supplement use for women who could become pregnant, and the subsequent major public health campaign, were shown to result in limited behavior change in the United States. This, together with the fact that 50% of pregnancies in the United States are unplanned, led to the fortification of staple foods with folic acid being mandated in January 1998. Fortification was also mandatory less than a year later in Canada (Dunlap et al., 2011). Data from the United States that included prenatal diagnoses of NTDs found that postfortification there was a 27% decline in NTDs (CDC, 2004) and for Canada, data similarly including prenatal NTD diagnoses, found a 46% reduction (De Wals et al., 2007).

In 1992, the U.K. Department of Health issued a recommendation that 400 μg of folic acid per day should be taken during the periconceptional period: before conception and throughout the first 12 weeks of pregnancy. This was increased to 5 mg per day for women with a risk of folate deficiency or previous NTD, and also for women with diabetes or epilepsy. An audit of women’s compliance with this advice found that only 3% of women in the study sample had taken folic acid supplements in the preconception period (Clark and Fisk, 1994). This resulted in a Health Education Authority (HEA) Campaign being launched in February 1996. Key components of this campaign were to increase awareness of the importance of taking folic acid periconceptionally, to educate health professionals and to promote voluntary fortification (Raats et al., 1998). An audit in 1996 to 1997 found that, although knowledge of the beneficial effects of folic acid had increased, compliance was still low (Neill et al., 1999). Despite these findings, a decision to introduce mandatory folic acid food fortification was not implemented in the U.K.

It is unclear whether NTD rates have declined in the U.K. post-1992, when the folic acid supplementation policy was introduced. Botto et al. (2005) reported that supplementation policies in several countries, including the U.K., did not seem to have influenced trends in NTDs up to 6 years after policy introduction. Busby et al. (2005) found that NTD prevalence in the U.K. and Ireland had fallen by 32%, but this continued a pre-existing downward trend in NTD frequency. Although it is similarly difficult to establish how much of the decline postfortification in the United States and Canada is due to a pre-existing reduction, the clear drop in NTD rates in both countries was matched by an increase in average red blood cell folate concentrations in the early years postfortification (CDC, 2007), supportive of a causal link. There is still no mandatory fortification policy in the U.K., or anywhere in Europe, so improvement in periconceptional knowledge and voluntary use of folic acid remains a high priority.

A recent systematic review reported that periconceptional folic acid use remains low in the U.K., with 48% of women taking folic acid periconceptionally as a best estimate, and only 21% as the worst (Stockley and Lund, 2008). Unintended pregnancy, age, socioeconomic status, and ethnic group were identified as important factors associated with low folic acid supplement use. It is argued that the factor most associated with low folic acid supplement use is unintended pregnancy, followed by age, socioeconomic status, and then ethnic group (Stockley and Lund, 2008). However, while the majority of U.K. studies have assessed folic acid knowledge and use in the U.K. with reference to socioeconomic status and age, very few have investigated the role of ethnicity.

The 2011 Office for National Statistics census data for England and Wales showed that 86% of usual residents are White (80% of total population were White British), 7.5% Asian/Asian British (2.5% Indian, 2% Pakistani, 1.5% Other Asian, 0.8% Bangladeshi, and 0.7% Chinese), and 3.3% Black/African (1.8% African, 1.1% Caribbean, and 0.5% Other Black). In London, 18.5% of the population is Asian/Asian British and 13.3% Black/African (ONS, 2012). The Health Survey for England conducted in 2004 found that obesity levels are higher in Pakistani, Black African, and Black Caribbean groups (Gatineau and Mathrani, 2011); poor diet, particularly for South Asians, and lower socioeconomic status, were indicated as possible reasons for this (Gatineau and Mathrani, 2011). It has also been shown that women with type 2 diabetes are more likely to come from ethnic minority groups (Macintosh et al., 2006). It is known that women who are obese or have diabetes have an increased risk of having an NTD-affected pregnancy (Macintosh et al., 2006; Waller et al., 2007).

Pre-1991 data from the North West Thames region in the U.K. (which includes parts of London) found that the incidence of NTDs (terminations were not included) in the Pakistani population in the U.K. was higher than in the European population. It is difficult to tell whether this was a true difference or due to the Pakistani population having fewer terminations (Chitty and Winter, 1989). Post-1992 data, in the “folic acid era,” suggested that the rate of NTD-affected pregnancies (including terminations) in the North West Thames region was higher in women of Pakistani or Indian origin (Michie et al., 1998). Preliminary data from the West Midlands congenital anomaly register also indicated a higher rate of NTD-affected pregnancies in Pakistani women (Tonks et al., 1995). Red cell folate concentrations were also lower in Indian and Pakistani women, but it is difficult to tell whether this was due to genetic mutations in folate metabolizing genes or due to a lower folic acid intake, or a combination of the two (Michie et al., 1998).

A study conducted on much earlier data (1960–84), before the folic acid era and during a period when very few terminations would have been carried out, found that the birth prevalence of spina bifida was significantly higher in Europeans than those of South Asian origin (Leck and Lancashire, 1995), indicating that lower folic acid intake is likely to be a greater contributor to the increased rate of NTD-affected pregnancies in Indian and Pakistani women in more recent years; this, however, would need to be substantiated with further research.

Approximately 40% of pregnancies are estimated to be unplanned in the U.K. (Dex and Joshi, 2004); therefore, women may have knowledge of the benefits of folic acid but do not take it because they are not planning to become pregnant. However, recent folic acid campaigns in the U.K., such as “Go Folic” (SHINE, 2012) have changed the focus from those planning pregnancy to those who might become pregnant (including women using contraception and actively trying not to get pregnant). Studies assessing women’s knowledge of the role of folic acid in NTD prevention, and their sources of information, are important for understanding why women do or do not use folic acid. Specifically for ethnic communities this is critical for identifying where health education campaigns should be targeted and/or why a fortification policy is so important. The current review, therefore, sought to evaluate how folic acid knowledge and periconceptional use for NTD prevention varies by ethnicity in the U.K.

## MATERIALS AND METHODS

### Study Selection

Medline, Embase, Pubmed, and Cochrane databases were searched by one reviewer as part of a wider literature review looking at NTDs in ethnic communities (using keyword and MESH search terms: neural tube defect* and ethnic*). Titles, abstracts, and full articles where necessary were scrutinized to identify U.K. studies that used post-1991 data: that is, following publication of the Medical Research Council’s vitamin study (MRC Vitamin Study Research Group, 1991). The full articles for all U.K. studies were downloaded, and studies that had assessed folic acid knowledge or periconceptional use in women of non-White or non-Caucasian ethnicity were included in the review, irrespective of study design. Referenced articles from extracted publications were also reviewed for any relevant studies.

### Study Evaluation

Papers that assessed knowledge of folic acid were evaluated for their quantification of how many women within different ethnic groups understood the benefits of folic acid, and their sources of information. Papers that studied folic acid use were evaluated for quantitative information on numbers of women in different ethnic communities who took folic acid in the periconceptional period, although some studies included data on preconceptional folic acid use only. All findings were evaluated in the context of individual studies’ strengths (e.g., large sample size for non-Caucasian participants) and limitations (e.g., possible responder bias).

### Meta-analysis

Preconceptional folic acid use in Caucasians was compared with non-Caucasians across all relevant studies using a random effects model, chosen because of expected between-study variation. Relevant studies were those in which preconceptional folic acid use had been quantified in different ethnic groups that could appropriately be categorized as Caucasian and non-Caucasian. The *metan* command was used in STATA (StataCorp, College Station, TX) to run the meta-analysis, which includes estimating a suitable summary statistic for each study, and then giving a weighted average of that statistic across studies. The results were summarized in a forest plot. The *metan* command also gives an estimate of heterogeneity across studies. The *metafunnel* command, which produces a graph with treatment effects from individual studies plotted on the x axis against a measure of study size on the y axis, was also used to investigate any publication or other bias in the meta-analysis.

## RESULTS

**Figure 1:**
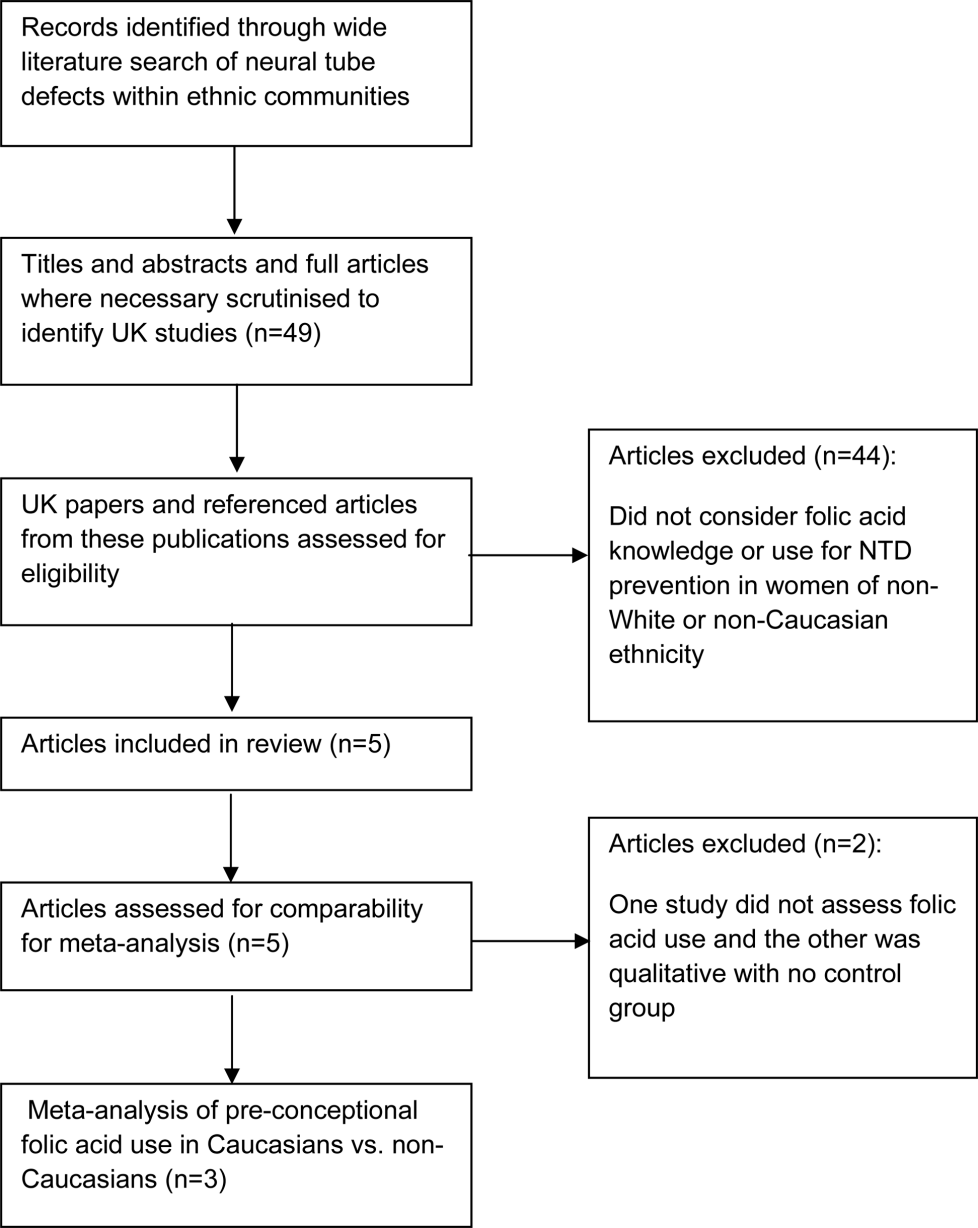
Flow diagram detailing process of selection of studies for inclusion in systematic review and meta-analysis. NTD, neural tube defect; UK, U.K.

From the wider literature review looking at NTDs in ethnic communities, the full articles for 49 papers were downloaded. Of these, five met the full inclusion criteria: an assessment of folic acid knowledge or use in those of non-White or non-Caucasian ethnicity using post-1991 U.K. data (Fig. 1). Study summaries are shown in Table 1 and specifically the breakdown of the number and proportion of participants in each study by ethnicity, is shown in Table 2.

**Table 1:** Study Summaries

Study	Study setting	Study population	Study design	Study period	Knowledge of folic acid benefits assessed?	Periconceptional folic acid use assessed?	Other Information
Krischer (1997)	2 GP practices (presumed Bristol due to author’s location but unspecified)	105 women from inner GP practice and 103 women from suburban GP practice; aged 15–40	Self-completion questionnaire	Inner city GP: Aug 1995 Suburban GP: Dec 1995	Yes	No	Women included whether or not they were attending antenatal appointments.
							Not stated how ethnicity was defined.
Jessa and Hampshire (1999)	GP antenatal clinics in Nottingham	13 pregnant British Pakistani women attending consecutive antenatal clinics	In-depth interview (in English, Urdu or Punjabi)	Not stated	Yes	Yes	Ethnicity was ascertained using names recorded on antenatal lists.
Howell et al (2001)	Antenatal clinics in Tower Hamlets, London	249 women (120 Bangladeshi and 100 white) attending for first antenatal appointment	Verbally administered questionnaire before appointment	October 1997-July 1998	Yes	Only pre-conceptional folic acid use	Self-described ethnic group.
Brough et al (2009)	Homerton Hospital antenatal clinic or 2 community clinics in Hackney, East London	402 pregnant women attending for first antenatal appointment	Researcher-led questionnaire	June 2002-May 2004	No	Yes. Before 6 weeks gestation was of particular interest.	Self-described ethnic group.
							Non-English speakers and those taking multi-vitamins (excluding folic acid and iron) excluded.
Lane (2011)	Three South Wales Hospitals’ antenatal clinics	386 pregnant women	Anonymous self-completion questionnaire	May 2010	Results were not broken down by ethnicity	Yes	Self-described ethnic group

**Table 2:** Breakdown by Ethnicity of the Number and Proportion of Participants in Each Study

No. of cases (% of total in each study)					
Ethnicity	Krischer, 1997	Jessa and Hampshire, 1999	Howell et al., 2001	Brough et al., 2009	Lane, 2011
White or Caucasian	White: 166 (80)		White: 100 (40)	Caucasian: 155 (39)	White: 307 (80)
Afro-Caribbean	15 (7)				
Asian	18 (9)			42 (10)	32 (8)
Pakistani		13 (100)			
Bangladeshi			120 (48)		
African				111 (28)	
West Indian				66 (16)	
Black					15 (4)
Chinese					2 (1)
Mixed race					5 (1)
Other/Unknown	9 (4)		29 (12)	28 (7)	25 (6)

The main reason for excluding papers was that they did not consider folic acid knowledge or use in those of non-White or non-Caucasian ethnicity. For example, in one of the excluded studies (Tedstone et al., 2008), minority ethnic participants were included but there were no specific results reported, broken down by ethnicity, of knowledge of the link between spina bifida and folic acid intake.

### Folic Acid Knowledge

Howell et al. (2001) reported ethnic differences in knowledge of folic acid benefits. Only 35% of Bangladeshi women had heard of the benefits of folic acid compared with 84% of white women. In contrast, Krischer (1997) reported that, in an inner-city general practitioner (GP) practice with a multi ethnic population, there were no key differences between ethnic groups in their knowledge of the link between folic acid and spina bifida. However, Krischer’s study was underpowered: of the two GP practices studied, only the inner city practice (105 participants) was multi-ethnic. Hence, numbers of participants were very small when different ethnic groups were compared. In Jessa and Hampshire’s (1999) study, none of the Pakistani women interviewed were aware of the link between folic acid and NTDs. The two remaining studies (Brough et al., 2009; Lane, 2011) did not report folic acid knowledge in participants with reference to ethnicity and no discrepancies could be inferred due to the relationship between knowledge and uptake. Interestingly, Brough et al. (2009) reported that only 16% of study participants stated they knew nothing about folic acid.

### Folic Acid Use

Four of the five studies reported data on folic acid use, with two (Howell et al., 2001; Brough et al., 2009) finding discrepancies between ethnic groups. Brough et al (2009) found ethnic differences in preconception use of folic acid: uptake was highest in Caucasians (19%) followed by Asians (12%), West Indians (8%), and Africans (5%). A further 23% of Caucasians had started to take folic acid postconception but before neural tube closure (6 weeks gestation cut-off in this study). Hence, it was concluded that 42% of all Caucasians had started to take folic acid before neural tube closure compared with 20% of African and 19% of West Indian and Asian mothers in the study. Brough and colleagues (2009) drew attention to two limitations of their study: it was not powered to investigate folic acid use and women taking multivitamins were excluded. The latter exclusion criterion could have biased the study toward participants of lower socioeconomic status, and potentially influenced the study’s ethnic make-up. In addition to discrepancies in folic acid usage between different ethnic groups, the study also found that folic acid was more likely to be used by mothers from a higher socioeconomic group or with higher education levels.

Howell et al. (2001) used univariate analysis to look at the relationship between ethnicity and folic acid intake, and multivariate analysis to control for the woman’s age, school leaving age, social class, parity, planned pregnancy, and whether women had heard about folic acid benefits. However, only a modest difference in outcome was found using these two analytical methods: white women were 5.7 times more likely to take folic acid preconception than Bangladeshi women in the univariate analysis, and 5.2 times more likely in the multivariate analysis.

In the qualitative study by Jessa and Hampshire (1999), none of the 13 Pakistani women interviewed increased their folic acid consumption in the periconceptional period.

The remaining study (Lane, 2011) found that marital status, income, and education were the factors that most influenced periconceptional folic acid use, but that ethnicity was not a significant factor. However, there were very few participants in ethnic groups other than White British, potentially weakening the argument that ethnicity was unimportant.

### Sources of Information on Folic Acid

The media (including magazines, newspapers, television, and radio) and health professionals (e.g., GPs and midwives) were the most commonly reported sources of information on folic acid (Krischer, 1997; Lane, 2011). British Pakistani women in Jessa and Hampshire’s (1999) study displayed great trust and respect for their GP and, although none of the women could recall their GP giving them any advice about folic acid, said they would only take folic acid supplements if their GP told them to. However, Lane (2011) reported that 81% of women who took folic acid because their GP or midwife advised them to do so, did not start taking the supplements until after they became pregnant.

### Meta-analysis

Data on preconceptional (as opposed to periconceptional) folic acid use in Caucasian and non-Caucasian women were included in three of the five studies (Howell et al., 2001; Brough et al., 2009; Lane, 2011), and so we used this information in the meta-analysis. In contrast, only two studies assessed knowledge of the benefits of folic acid, broken down by ethnicity, and it was judged inappropriate to carry out a meta-analysis in this case, owing to lack of data.

**Figure 2:**
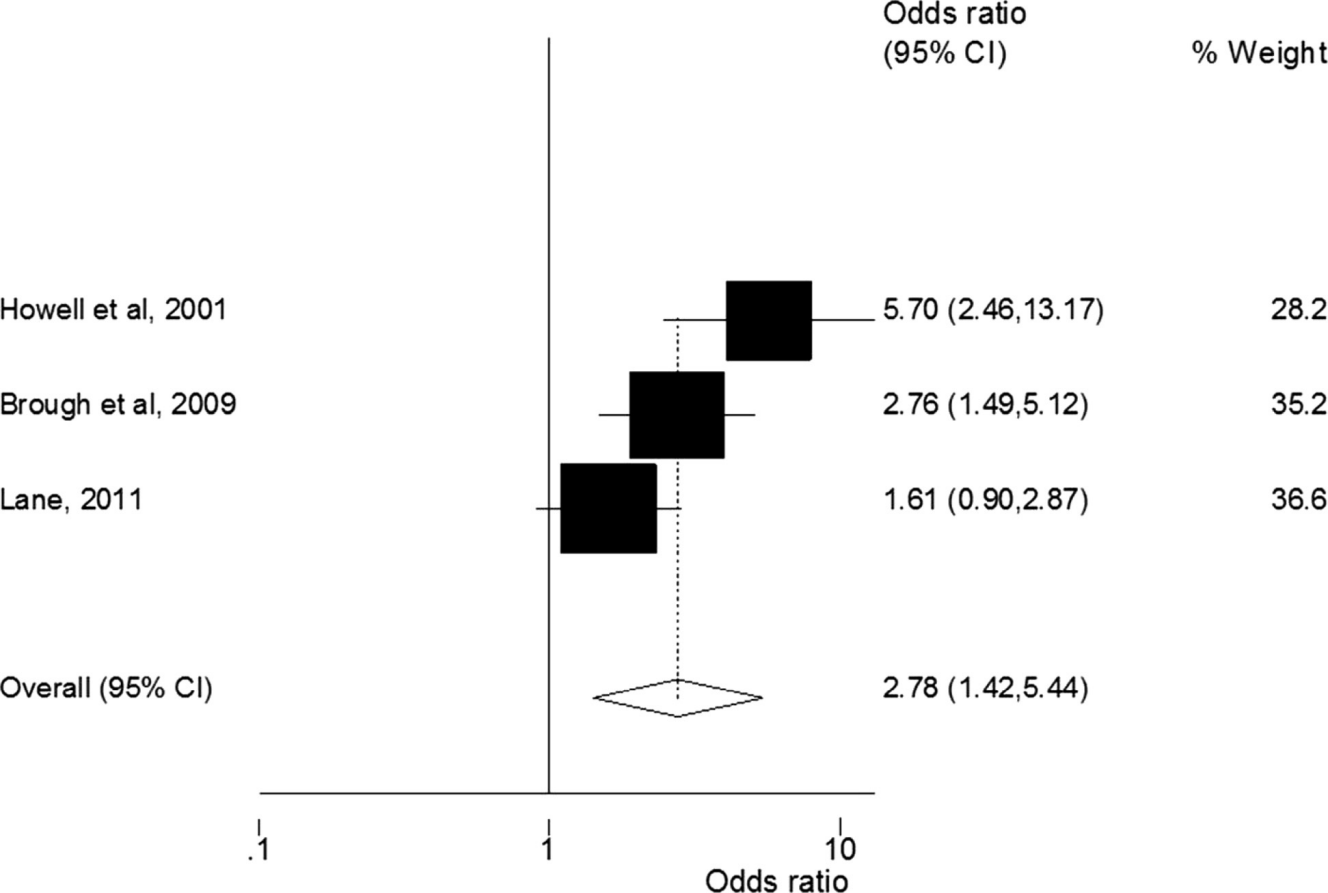
Meta-analysis of preconceptional folic acid use in Caucasians compared with non-Caucasians. The odds ratio for each individual study is given as well as an overall odds ratio across the three studies. Weighting of each study was determined according to the random effects model used for the meta-analysis.

Caucasians were compared with non-Caucasians as the contributing studies differed in their ethnic mix of participants and one study did not break ethnicity down any further than Caucasian British and Other (Lane, 2011). The meta-analysis across the three studies, using a random effects model, shows that Caucasians are almost three times as likely to take folic acid before conception as non-Caucasians in the U.K. (95% confidence interval: 1.42, 5.44) (Fig. 2). A random effects model was appropriately chosen (between study variation is allowed) as the analysis showed evidence of heterogeneity across studies that was of borderline significance (*p* = 0.049). Therefore, although Lane (2011) found no significant difference in preconceptional folic acid use between Caucasians and non-Caucasians, when the results from the three studies are pooled, there is a clear treatment effect. Investigations for bias in the meta-analysis found that no study unduly swayed the final treatment effect.

## DISCUSSION

In this study, we aimed to evaluate how folic acid knowledge and periconceptional usage for NTD prevention varies by ethnicity in the U.K. Considering previously published evidence, five studies met the inclusion criteria: to have assessed folic acid knowledge and/or periconceptional folic acid use in participants, including women of non-Caucasian ethnicity. A synthesis of folic acid usage data from three studies, in a meta-analysis, shows that Caucasians are almost three times more likely to have taken folic acid before conception than non-Caucasians. This study has been checked against PRISMA guidelines and meets accepted standards.

Studies were selected for our review by scrutiny of the wider literature on NTDs in ethnic communities. The aim was to ensure that all studies providing information on folic acid knowledge or use in the periconceptional period, in Caucasian and non-Caucasian communities, were captured. Due to time constraints, only one reviewer made determinations. However, the effectiveness of our scrutiny method is indicated by the fact that we identified all studies related to ethnicity included in Stockley and Lund’s (2008) publication. This series of systematic reviews concluded that ethnic group is one of the factors associated with low use of folic acid supplements.

Ethnicity was self-described in all studies included in the meta-analysis, although both Brough et al. (2009) and Lane (2011) combined ethnic groups for analytical purposes. The study periods were diverse between the three studies, which could have acted as a potential confounder. However, all were within the post-1991 “folic acid era” and, because folic acid use has been consistently low since folic acid supplementation policies were implemented (Neill et al., 1999; Stockley and Lund, 2008), this argues that variations in the three study periods are unlikely to have markedly affected findings. All studies included in the meta-analysis were conducted in antenatal clinics, specifically during the first antenatal visit for the Howell et al. (2001) and Brough et al. (2009) studies, although this was not clearly stated in Lane’s (2011) study. This is important as completing the questionnaire at the first antenatal visit, at the end of the period when folic acid supplementation is recommended (12 weeks), is likely to have resulted in clearer recall.

Studies included in the present review were conducted in diverse areas throughout the U.K. Only Howell et al. (2001) and Brough et al. (2009) included participants from the same area: East London. Lane’s (2011) study was conducted in South Wales where the ethnic make-up is different. Combining these three studies in the meta-analysis, therefore, could have introduced variation due to geographical region within the U.K. that was sampled.

Comparison in the meta-analysis was based on a broad ethnic distinction: Caucasian versus non-Caucasian. This is certainly a limitation of the data currently available, as it does not allow conclusions to be drawn about specific non-Caucasian groupings. It did, however, allow us to focus specifically on whether U.K. Caucasian (i.e., largely indigenous) women are more or less likely to take folic acid preconceptionally than non-Caucasians (including many migrant families).

There are indications that different non-Caucasian groups may differ in their knowledge and use of folic acid in the periconceptional period. Only two of the five studies included in the review considered both knowledge and use of folic acid in the periconceptional period, broken down by ethnicity (Jessa and Hampshire, 1999; Howell et al., 2001). Both studies reported low use of folic acid that mirrored a lack of knowledge of folic acid in women of South Asian origin. Brough et al. (2009) found that preconceptional use of folic acid was even lower in West Indians and Africans than Asians. However, no firm conclusions can be made on the basis of the published data, owing to the small number of studies and generally low participant numbers included.

The sources of information on folic acid that women reported in the studies suggest some potentially important conclusions. Jessa and Hampshire’s (1999) qualitative study highlighted a clear lack of prepregnancy advice on folic acid being given by the people the Pakistani women trusted most: their GPs. This is supported by a study showing that, compared with non-Asians, Asians are less concerned about the availability of over the counter medicines, preferring to consult their GP to obtain medication (Rashid et al., 1996). It would be important to establish the effectiveness, therefore, of promoting folic acid information in South Asian communities through South Asian media and community centers, as recommended by Jessa and Hampshire (1999), given that many women in this study said they would only take folic acid supplements if their GPs advised them to.

Understanding why women do, or do not, take folic acid in the periconceptional period is essential to inform health education campaigns that are likely to have the greatest impact. Indeed, this was a key aim of Lane’s (2011) study, as a literature search had not identified any British studies that specifically investigated this question. While Lane’s findings were not broken down by ethnicity, discovering pregnancy after 12 weeks of gestation was the reason given most often (34%) for not taking folic acid, presumably indicating the pregnancy was unplanned.

Four of the five papers reviewed in the present study (Krischer, 1997; Jessa and Hampshire, 1999; Brough et al., 2009; Lane, 2011) argued for food fortification as the most effective method of improving folate intake. Three of these studies (Krischer, 1997; Jessa and Hampshire, 1999; Brough et al., 2009) argued that specifically ethnic minority foods such as wheat and chapatti flour would need to be fortified to ensure ethnic communities benefit from fortification.

Research has been conducted in the United States to assess the impact of fortification on different ethnic communities. With respect to red blood cell folate levels, non-Hispanic whites had the highest prefortification levels followed by Hispanics, then non-Hispanic blacks. A study comparing red blood cell folate levels before and after fortification found that, despite absolute disparities in red blood cell folate levels decreasing, relative differences actually increased between non-Hispanic whites and non-Hispanic blacks, although there was no change in the relative difference between non-Hispanic whites and Hispanics (Dowd and Aiello, 2008). Twice as many non-Hispanic whites (43.9%) take folic acid supplements as Hispanics (20.8%) and non-Hispanic blacks (19.3%) (Yang et al., 2007). For Hispanics, improved folic acid supplement use needs to be promoted, in addition to fortification of corn flour, a staple constituent of the Hispanic diet that was not included in the mandatory fortification policy in the United States, to improve folic acid intake (Dunlap et al., 2011).

### Conclusions

The results of the meta-analysis showed that Caucasians are more likely to take folic acid preconception than non-Caucasians. From the wider review, Asians, specifically South Asian women, appear to have less knowledge and lower usage of folic acid. However, furthermore, more rigorous, research is required to make any firm conclusions about diversity among non-Caucasian population groups. An understanding of why women do, or do not, take folic acid is critical for the effective targeting of health education campaigns and there is a clear need for more qualitative research in this area. The qualitative study included in this review accentuated the importance of GPs providing accurate prepregnancy health information as part of routine practice, as many appear to be giving advice to women about folic acid when it is too late for their current pregnancy. Conclusions from the current study, together with other important evidence from the U.K., United States, and Canada, emphasize that carefully targeted, innovative education campaigns to improve supplement use within ethnic communities should be implemented in combination with a mandatory fortification policy, including the fortification of ethnic minority foods, to ensure as many NTDs are prevented as possible.
